# Case-control study of tobacco smoke exposure and breast cancer risk in Delaware

**DOI:** 10.1186/1471-2407-8-157

**Published:** 2008-06-02

**Authors:** Dana E Rollison, Ross C Brownson, H Leroy Hathcock, Craig J Newschaffer

**Affiliations:** 1 Division of Cancer Prevention & Control, H Lee Moffitt Cancer Center & Research Institute; Tampa, FL, USA; 2 Department of Community Health and Prevention Research Center, Saint Louis University School of Public Health, St Louis MO, USA; 3 Delaware Division of Public Health, Dover, DE, USA; 4 Department of Epidemiology and Biostatistics, Drexel University School of Public Health, Philadelphia, PA, USA; 5 Department of Epidemiology, Johns Hopkins Bloomberg School of Public Health, Baltimore, MD, USA

## Abstract

**Background:**

Tobacco smoke exposure may be associated with increased breast cancer risk, although the evidence supporting the association is inconclusive. We conducted a case-control study in Delaware, incorporating detailed exposure assessment for active and secondhand smoke at home and in the workplace.

**Methods:**

Primary invasive breast cancer cases diagnosed among female Delaware residents, ages 40–79, in 2000–2002 were identified through the Delaware cancer registry (n = 287). Delaware drivers license and Health Care Finance Administration records were used to select age frequency-matched controls for women <65 and ≥ 65, respectively. Detailed information on tobacco smoke exposure was obtained through telephone interviews.

**Results:**

A statistically significant increased risk of breast cancer was observed for ever having smoked cigarettes (odds ratio = 1.43, 95% confidence interval = 1.03–1.99). However, there was no evidence of a dose-response relationship between breast cancer risk and total years smoked, cigarettes per day, or pack-years. Neither residential nor workplace secondhand smoke exposure was associated with breast cancer. Recalculations of active smoking risks using a purely unexposed reference group of women who were not exposed to active or secondhand smoking did not indicate increased risks of breast cancer.

**Conclusion:**

These findings do not support an association between smoking and breast cancer.

## Background

More than 180,000 cases of breast cancer are expected to be diagnosed in the U.S. in 2008 [1]. While several reproductive and genetic risk factors for breast cancer have been well established in the epidemiologic literature, these factors account for less than half of all breast cancer and are not modifiable [2]. Exposure to tobacco smoke is a potentially modifiable factor that may be associated with increased breast cancer risk, although evidence supporting the association is inconclusive.

While some epidemiologic studies have observed associations between cigarette smoking and breast cancer, more studies have not [3,4]. Most studies of secondhand smoke exposure suggested no association with breast cancer risk [5-17], although a few have observed increased breast cancer risk among women exposed to secondhand tobacco smoke [18-21], particularly among premenopausal women [22] and women with exposure prior to first full-term birth [23,24]. Notably, studies that measured detailed exposures to both active and secondhand tobacco smoke indicate that risk associated with active smoking may have been masked in earlier studies because women exposed to secondhand tobacco smoke were included in the reference groups [18-20,24-27]. A recent meta-analysis concluded that studies with more thorough assessment of exposure to tobacco smoke tended to support an association between breast cancer and both active and secondhand tobacco smoke, whereas studies with cruder exposure assessment were less likely to observe associations [28]. Here we report on a case-control study in Delaware, incorporating detailed exposures to active and secondhand smoke assessed both at home and in the workplace during critical periods in a woman's reproductive life.

## Methods

### Study population

Breast cancer cases were identified through the Delaware Cancer Registry, a statewide, population-based cancer registry maintained by the Delaware Division of Public Health. Cases were defined as female residents of Delaware, ages 40–79, diagnosed with microscopically-confirmed incident primary invasive breast cancer (International Classification of Diseases, 10^th ^revision (ICD-0) code 174) in 2000–2002. Cases were excluded from the study if they had a prior history of invasive breast cancer, lived in an institutional setting, or did not have a telephone. Physician approval was required prior to contacting cases. Potential participants were then mailed a letter and contacted by telephone 1–2 weeks later. Of the 1,076 potential cases identified through the Delaware Cancer Registry, physician permission was obtained to contact 617, of whom 217 could not be located or were not eligible for the study. Of the remaining 400 eligible cases, 28% refused to participate in the study, resulting in a final sample size of 287 cases. As compared to the cases who participated in the study, cases who were not contacted or refused to participate were somewhat less likely to be White, while no differences were observed between the groups by county or stage at diagnosis.

Controls were selected using two public databases: Delaware motor vehicle drivers license records for women <65 years and records maintained by the Health Care Finance Administration (HCFA), currently known as the Center for Medicare and Medicaid Services, for women 65+ years. Controls were frequency-matched to cases on age within 10-year groups. Telephone numbers were obtained using reverse telephone directories, and letters were mailed to women with phone number matches, informing women of their selection into the study and the phone call that they should anticipate. Potential controls were then called 1–2 weeks after letters were mailed, and of those eligible controls contacted, 46% participated.

Power calculations performed during the design phase of the study indicated that 300 breast cancer cases and 300 controls were needed in order to achieve 80% power to detect an odds ratio between active smoking and breast cancer of 1.6 at a significance level of p < 0.05, assuming the prevalence of exposure in controls was 47%, the proportion of women of similar ages as the proposed controls who reported ever smoking in the Delaware Behavioral Risk Factor Surveillance Survey [29].

### Data collection

In accordance with the study protocol, which was approved by the human subjects review boards at the Johns Hopkins Bloomberg School of Public Health, the Delaware Department of Health and Social Services, and the University of Delaware, informed consent was completed by phone. Trained interviewers administered a detailed questionnaire with smoking questions modeled after the Missouri Women's Health Study [30,31]. Each woman who had ever smoked (i.e. smoked at least 100 cigarettes in her lifetime) answered questions about the age she first smoked cigarettes, how many cigarettes she smoked per day, whether she smoked cigarettes that were filter versus non-filter, menthol versus non-menthol, and regular versus low-tar, if she usually inhaled smoke into the chest, and if in addition to cigarettes, she used any other type of tobacco product on a regular basis, including pipes, cigars, cigarillos, snuff, and chewing tobacco. If any of these characteristics of her smoking habits changed since she started smoking, then the age at the time of the change was recorded, and the same characteristics were assessed for the next smoking period.

Secondhand smoking in childhood (<18 years) and adulthood was based on enumeration of smokers living in the participant's household. Then, for each smoker, the number of packs, cigars or pipefulls smoked per day were recorded, in addition to the hours/day the participant was exposed. As with active smoking, new data were collected any time any aspect of exposure changed. Occupational exposure to secondhand smoke involved enumeration of jobs with exposure and, for each job, reporting of length of employment and average daily hours of exposure. Subjects also provided a subjective rating of exposure intensity (light, moderate, heavy) for each job. Other questions on demographics, reproductive history, family history, exogenous hormone use, alcohol consumption, physical activity, sleeping habits, meat cooking and consumption, and other dietary factors were also included.

### Statistical methods

Categorical variables were compared between cases and controls using the chi-square test or the Mantel-Haenzsel chi-square test for trend. Total years smoked were calculated by summing the lengths of each reported period of smoking. Average cigarettes per day were calculated by weighting the number of cigarettes smoked per day in each period by the number of years in that period, divided by the total number of years. Total pack-years were calculated as the product of total years and average cigarettes per day.

Secondhand smoking analyses were restricted to women who smoked less than 100 cigarettes in their lifetime. Total years of secondhand smoke exposure were calculated by summing years across all periods for which women reported residential secondhand smoke exposure. To facilitate comparison with findings from a previous study of detailed secondhand smoke exposure measures [18], a smoker-year was defined for each period of residential secondhand smoke exposure as the product of the number of years in the period and the number of smokers who smoked in the household during that period. Total smoker-years were calculated as the sum of smoker-years across periods. Pack-years were defined as the product of cigarettes smoked per day for a given smoker in a given period and the number of years in that period. Pack-years were summed across smokers for each period and then across periods to generate a total pack-year measure. Weighted pack-years were defined as the product of cigarettes smoked per day for each smoker in a given period, the number of hours the woman was exposed to that smoker's smoke each day in that period, and the times the number of years in a period, divided by 18 waking hours in a day. This measure was summed across smokers in each period and across periods to obtain a total weighted pack-years measure. Both years and smoker-years were calculated for all types of tobacco products combined. Average cigarettes per day, pack-years and weighted pack-years were calculated for cigarette exposure only. All secondhand smoke exposure measures were calculated separately for childhood (exposure prior to age 18) and adulthood and summed to obtain a measure of lifetime secondhand smoking.

Associations between tobacco smoke exposure and breast cancer were estimated by using unconditional logistic regression. For initial active smoking analyses, never active smokers (women who never smoked 100 cigarettes) were the reference group. For secondhand smoke exposure, never active smokers unexposed to secondhand smoke comprised the reference group. Additional active smoking analyses were conducted, using three more restrictive reference groups based on secondhand exposure data; those with: 1) zero years of lifetime residential secondhand smoke exposure and zero years of occupational secondhand smoke exposure; 2) ≤ 10 years of lifetime residential secondhand smoke exposure, ≤ 10 years of moderate-level occupational secondhand smoke exposure, and ≤ 10 years of heavy-level occupational secondhand smoke exposure; or 3) zero years of residential secondhand smoke exposure before age of 18. Women missing data for any smoker in any period were coded to missing for the corresponding summary variable.

Initial regression models included age and menopausal status, given their strong associations with breast cancer. Additional potential confounders were tested individually. If the risk estimate associated with smoking changed by 10 percent in either direction, that variable was included in a full model. If the smoking variables were multilevel, then a factor was included in the final model if it affected 1 out of 2 or 2 out of 3+ levels. Potential confounders were assessed separately for active and passive tobacco smoke exposure. Separate models were also constructed for pre- and postmenopausal women.

## Results

Selected characteristics of cases and controls are presented in Table 1. Controls were disproportionately premenopausal and college-educated, but similar to cases on other characteristics. Ever-active smoking was associated with a statistically significant increased risk of breast cancer (OR = 1.43, 95% confidence interval (CI) = 1.03–1.99) (Table 2). However, there was no evidence of a dose-response trend in breast cancer risk for total years smoked, average number of cigarettes per day or total pack-years. Ever active smoking prior to the age of 18 was associated with increased risk, but the magnitude of the association was not greater than that for ever smoking overall, and there was no dose response for years smoked prior to age 18. Active smoking before first live birth was not associated with increased risk of breast cancer (Table 2). The risk of breast cancer associated with ever having smoked cigarettes was similar for premenopausal women (30 cases, 65 controls; OR = 1.53, 95% CI = 0.60–3.95) and postmenopausal women (256 cases, 246 controls; OR = 1.36, 95% CI = 0.95–1.94). Analyses limited to different types of cigarettes or inhalation levels did not reveal additional associations (data not shown).

**Table 1:** Selected characteristics of breast cancer cases and controls, Delaware, 2000–2002

	cases (n = 287)		controls (n = 311)		
			
Characteristic	n	%	n	%	p-value^a^
Age at interview (years)					
40–49	53	18.5	63	20.3	
50–59	74	25.8	94	30.2	
60–69	87	30.3	77	24.8	
70–80	73	25.4	77	24.7	0.32
Race					
White	264	92.0	294	94.5	
Black/Other	23	8.1	17	5.5	0.21
Education^b^					
<12 grades	30	10.5	14	4.5	
12 grades	114	39.9	108	37.8	
some college	108	37.8	148	47.6	
post-college	34	11.9	41	13.2	< 0.01
Menopausal status					
premenopausal	30	10.5	65	20.9	
postmenopausal	257	89.6	246	79.1	< 0.01
Body mass index (kg/m2)^b^					
15–24	107	37.3	122	39.6	
25–29	102	35.5	114	37.0	
30–88	78	27.2	72	23.4	0.56
Age at menarche (years)^b^					
8–12	140	49.3	142	45.8	
13–18	144	50.7	168	54.2	0.39
Number of Live Births^b^					
nulliparous^c^	44	15.3	35	11.3	
1–2	128	44.6	145	46.8	
3–4	97	33.8	112	36.1	
5–11	18	6.3	18	5.8	0.52
Age at 1st live birth (years)^b^					
< 30	219	90.1	242	88.0	
30+	24	9.9	33	12.0	0.44
Oral contraceptive use					
Never	120	41.8	110	35.4	
Ever	167	58.2	201	64.6	0.11
Other hormone use^b^					
Never	153	53.3	161	51.9	
Ever	134	46.7	149	48.1	0.74
Family History of Breast Cancer^b,c^					
No	220	77.7	257	82.9	
Yes	63	22.3	53	17.1	0.11
Alcohol consumption					
< 12 drinks in lifetime	37	12.9	49	15.8	
≥ 12 drinks in lifetime, but never had ≥ 1 drink per mo. for ≥ 6 mos	87	30.3	85	27.3	
drank ≥ 1 drink per mo. for ≥ 6 mos	163	56.8	177	56.9	0.52

^a ^p-values based on chi-square test; p-values based on Mantel-Haenszel chi-square test for trend for the following variables: age, education, BMI, and age at first live birth

^b ^Numbers do not always sum to total number of cases and/or controls due to missing information

^c ^Includes 9 cases and 10 controls who reported 1+ pregnancies

^d ^Family history of breast cancer defined as mother or sister ever diagnosed with breast cancer

**Table 2:** Active smoking and breast cancer among cases and controls, Delaware, 2000–2002

	cases		controls		odds ratio^a^	95% confidence interval^a^
				
Active smoke exposure	n	%	n	%		
Ever smoked 100 cigarettes						
No	124	43.2	161	51.8	1.00	reference
Yes	163	56.8	150	48.2	1.43	1.03–1.99
Total years smoked cigarettes^b^						
<10	23	8.0	26	8.4	1.36	0.72–2.55
10–19	27	9.4	33	10.6	1.13	0.64–2.01
20–29	34	11.9	26	8.4	1.80	1.02–3.20
30–39	36	12.6	32	10.3	1.31	0.76–2.25
40–49	27	9.4	21	6.8	1.50	0.79–2.82
50–62	15	5.2	12	3.9	1.21	0.52–2.84
Average number of cigarettes/day^b^						
<10	58	20.5	49	15.9	1.60	1.01–2.53
10–19	67	23.7	58	18.8	1.53	0.99–2.35
20–29	25	8.8	29	9.4	1.12	0.61–2.04
30–68	9	3.2	12	3.9	0.84	0.34–2.08
Total pack-years^b^						
<5	37	13.1	36	11.7	1.45	0.85–2.45
5–9	19	6.7	18	5.8	1.65	0.81–3.35
10–19	37	13.1	26	8.4	1.83	1.04–3.23
20–29	28	9.9	29	9.4	1.24	0.69–2.24
30–39	10	3.5	12	3.9	1.06	0.44–2.56
40–49	16	5.7	9	2.9	1.92	0.81–4.55
50–102	12	4.2	18	5.8	0.75	0.34–1.65
Years smoked at or before age 18^c^						
<5	93	32.4	91	29.3	1.34	0.91–1.96
5–10	16	5.6	15	4.8	1.33	0.62–2.85
Years smoked, before first live birth^d^						
Never smokers	108	37.6	138	44.4	1.00	reference
<5	38	13.2	36	11.6	1.25	0.73–2.13
5–9	59	20.6	55	17.7	1.37	0.87–2.16
10–14	12	4.2	27	8.7	0.69	0.33–1.45
15–39	11	3.8	9	2.9	1.99	0.76–5.18

^a ^adjusted for age, education, and menopausal status

^b ^Numbers do not always total due to missing data

^c ^Excludes women who smoked only after age 18

^d ^Excludes nulliparous women and those who smoked only after the birth of their first child

Among never active smokers, secondhand smoke exposure in the home during childhood was not associated with breast cancer risk, whether exposure was measured in total years, smoker-years, pack-years or weighted pack-years (see additional file 1). Weighting pack-years by the number of hours exposed to secondhand tobacco smoke each day did not result in any considerable differences in breast cancer risks (see additional file 1). Similarly, secondhand smoke exposure in the home during adulthood was not associated with breast cancer risk, regardless of the exposure metric used (OR = 0.98, 95% CI = 0.58–1.64). Combining childhood and adulthood exposures, no increased risks were observed with 45 or more pack-years of lifetime residential secondhand smoke. Secondhand smoke exposure at work was not associated with increased breast cancer risk, even when considering 20–45 years of exposure duration and "heavy" self-rated intensity of exposure (Table 3).

**Table 3:** Secondhand smoke exposure at work and breast cancer among non-smoking cases and controls from Delaware

Secondhand smoke exposure at work^a^	cases		controls		odds ratio^b^	95% confidence Interval^b^
				
	n	%	n	%		
Never employed or						
not exposed at work	60	48.4	69	43.4	1.00	Reference
Any secondhand smoke exposure at work	64	51.6	90	56.6	0.80	0.49–1.32
Number of years employed at a job with secondhand smoke exposure						
< 10	27	21.8	48	30.1	0.66	0.35–1.23
10–19	24	19.4	27	17.0	1.02	0.52–2.00
20–45	13	10.5	15	9.4	0.86	0.35– 2.07
Number of years employed at a job with **light **secondhand smoke exposure						
< 10	17	13.7	27	17.0	0.96	0.46–1.97
≥ 10	13	10.5	13	8.2	1.14	0.47– 2.78
Number of years employed at a job with **moderate **secondhand smoke exposure						
< 10	11	8.9	16	10.1	0.70	0.29– 1.68
≥ 10	17	13.7	22	13.8	1.01	0.49– 2.09
Number of years employed at a job with **heavy **secondhand smoke exposure						
< 10	5	4.0	8	5.0	1.02	0.31– 3.37
≥ 10	7	5.6	7	4.4	1.07	0.35– 3.30

^a ^2 non-smoking controls were missing data on exposure to smoke at work

^b ^Adjusted for age, menopausal status, body mass index (<25, 25–29, 30+), age at menarche (<12 vs. 12+), age at first live birth (nulliparous, <30, 30+), oral contraceptive use (ever vs. never), other hormone use (ever vs. never), family history of breast cancer (yes vs. no), alcohol consumption (ever drank 12 drinks in lifetime vs. had 12 drinks in lifetime but never had 1+ drink per month for 6+ months vs. ever had 1+ drink per month for 6+ months); data for adjustment factors was missing for 4 cases and 3 controls

In active smoke exposure re-analyses, only 13 cases and 14 controls comprised the most stringent reference group of ever active smokers with no secondhand smoke exposure at home or at work during their lifetimes (Table 4). Using this reference group, ever having smoked cigarettes was not statistically significantly associated with breast cancer (OR = 1.21, 95% CI = 0.55–3.32), after adjustment for age and menopausal status. There was no evidence of a dose-response relationship between smoking and breast cancer risk using this reference group, nor were exposures before 18 or before first live birth associated with breast cancer (Table 4). As mentioned, to increase the sample size of the reference group, two less stringent definitions were considered, but no statistically significant associations were observed using either of these alternative reference groups (Table 4).

**Table 4:** Active smoking and breast cancer among cases and controls from Delaware, using women never exposed to active or secondhand smoking as the reference group

Active cigarette smoking exposure	Unexposed definition # 1^a^				Definition # 2		Definition # 3	
			
	cases	controls	OR^b^	95% CI^b^	OR^b^	95% CI^b^	OR^b^	95% CI^b^
Unexposed to active and secondhand smoke^a^								
Definition # 1	13	14	1.00	reference				
Definition # 2	30	37			1.00	reference		
Definition # 3	41	46					1.00	reference
Ever active smoker	163	150	1.21	0.55–2.66	1.33	0.77–2.27	1.23	0.76–1.98
Years of smoking^b^								
< 20	50	59	0.98	0.42–2.31	1.10	0.59–2.05	1.03	0.58–1.82
20–39	70	58	1.31	0.57–3.02	1.45	0.80–2.63	1.35	0.77–2.36
≥ 40	42	33	1.35	0.55–3.32	1.48	0.74–2.96	1.31	0.69–2.47
Cigarettes per day^c^								
< 10	58	49	1.31	0.56–3.08	1.45	0.78–2.71	1.33	0.75–2.35
10–19	67	58	1.30	0.56–3.00	1.44	0.79–2.63	1.33	0.76–2.32
≥ 20	34	41	0.90	0.37–2.18	0.99	0.51–1.94	0.91	0.49–1.69
Pack-years^c^								
< 10	56	54	1.20	0.51–2.81	1.34	0.72–2.48	1.25	0.70–2.21
10–29	65	55	1.30	0.56–3.01	1.43	0.78–2.63	1.32	0.75–2.31
≥ 30	38	39	1.03	0.43–2.49	1.13	0.58–2.20	1.02	0.55–1.89
Any exposure before age 18	109	106	1.13	0.51–2.52	1.27	0.73–2.21	1.18	0.71–1.95
Any exposure before first live birth	120	127	1.06	0.48–2.35	1.18	0.68–2.04	1.08	0.66–1.76

^a ^Women unexposed to secondhand smoke were defined as follows: Definition #1: Zero years lifetime residential and occupational exposure; Definition #2: ≤ 10 years lifetime residential exposure, ≤ 10 years of moderate-level occupational exposure and ≤ 10 years heavy-level occupational exposure; Definition #3: Zero years of residential exposure before age 18

^b ^OR, odds ratio; CI, confidence interval; adjusted for age and menopausal status

^c ^Data missing on total years smoked for 1 case, and cigs/day and pack-years for 4 cases and 2 controls

## Discussion

Overall, our findings do not support exposure to active or secondhand tobacco smoke as a risk factor for breast cancer. A statistically significant increased risk of breast cancer was observed for ever having smoked at least 100 cigarettes in one's lifetime. However, the magnitude of this risk was modest (OR = 1.43) and decreased rather than increased with use of more stringent reference groups excluding those exposed to secondhand smoke. Additionally, there was no evidence of a dose-response relationship between breast cancer risk and total years smoked, cigarettes per day, or pack-years. Residential exposure to secondhand smoke was not associated with breast cancer risk, whether the exposure occurred in childhood or adulthood. No increased risk of breast cancer was associated with heavy exposure to secondhand smoke at work. No large differences in breast cancer risk associated with secondhand smoke were observed by menopausal status.

Our findings for secondhand smoke are consistent with the 2002 International Agency for Research on Cancer Monograph on Tobacco Smoking and Tobacco Smoke, which stated that existing evidence was inconsistent and did not support a causal association between passive smoking and breast cancer [32]. In contrast, the more recent report published by the California Environmental Protection Agency concluded that the weight of the evidence was consistent with a causal association between secondhand smoke exposure and breast cancer diagnosed in women younger than age 50 who are mostly premenopausal [33]. Premenopausal women accounted for only 11 percent of the breast cancer cases included in the present study, thus, power to assess differences in risk by menopausal status was limited.

Although a number of previous studies report no association between active smoking and breast cancer risk (reviewed in [3,4]), these tended to use less intensive exposure assessment and include all non-smokers in the reference group. Our study, which meets criteria for high quality exposure assessment as outlined in a recent meta-analysis [28], found no association when the reference group excluded women with secondhand smoke exposure in contrast to other past studies meeting these criteria [19,20,24-28]. Use of a more stringent reference group did not increase the smoking risk ratios in part because of the limited number of women who had no active or passive smoke exposure and the lack of an association between secondhand smoking and breast cancer in this sample. This association was not seen despite collection of extensive secondhand smoke exposure histories, including the number of smokers in the home, the amount smoked by each smoker, and the hours per day of exposure to others' smoke, both in childhood and adulthood, in addition to secondhand smoke exposures at the workplace. It is highly unlikely that any major sources of secondhand smoke were missed in this study.

Some have suggested that there may be a critical period for exposure to active and/or secondhand smoking at early ages, prior to the birth of a first baby when cellular differentiation within the breast is completed [23,24]. In our study, women who reported smoking prior to age 18 or prior to their first live birth were not more likely to have breast cancer than those who did not actively smoke during these periods, even after restriction of the reference group to women who were not exposed to secondhand smoke in childhood. Our findings are consistent with a recent meta-analysis of 12 studies [6,11,14,19,23,24,34-38] that calculated a pooled risk estimate of 1.07 (95% CI: 0.72, 1.00), concluding that active smoking prior to first live birth is not associated with breast cancer [38]. Results from the California Teacher's Study recently suggested that only long duration tobacco smoke exposure prior to the first live birth increased breast cancer risk [39]. We did not observe differences in breast cancer risk associated with 30 or more pack-years of active smoking among those with five or more years of pre-partum tobacco smoke exposure (OR = 1.78, 95% CI = 0.66–4.85) versus those with less than five years of exposure (OR = 0.94, 95% CI = 0.50–1.77). However, among nulliparous women in this study, 28 of 44 cases and 12 of 35 controls reported ever smoking 100 cigarettes in their lifetime (OR = 3.34, 95% CI = 1.31–8.48), and when the reference group was restricted to women with zero years of residential secondhand smoke exposure before the age of 18, the risk of breast cancer associated with ever smoking 100 cigarettes became even stronger (OR = 8.11, 95% CI = 1.22–53.92), but was based on only 15 cases and 10 controls. Larger studies should attempt to replicate these small subgroup findings, since nulliparous women are unique in that their entire life of exposure is "prepartum" and may represent a susceptible subgroup to particular exposures related to increased breast cancer risk.

The statistically significant increased risk of breast cancer we observed for ever smoking juxtaposed with the lack of a dose-response relationship observed for any measure of frequency or duration suggests that the former association may be due to confounding. In a recent cross-sectional analysis from the California Teacher's Study [40], active smoking was associated with increased alcohol consumption, nulliparity, and among parous women, and later age at first birth. These factors were systematically assessed as potential confounders in the present study, and none affected the smoking odds ratios by 10 percent or greater. However, the observed overall association between ever smoking and breast cancer could be confounded by unknown factors. Recall bias may also explain the statistically significant increased risk of breast cancer observed with ever smoking, if the controls were less likely to remember past exposures than the cases. Alternatively, the association could simply be due to chance or could be due to differential nonparticipation among controls that were active smokers. While control non-participation rates were comparable to those from other breast cancer case-control studies [41,42] they were of a magnitude where differential participation would likely influence odds ratio estimates. That said, one case-control study with similar control non-participation rate still observed increased risk among long-term smokers compared to non-smokers [42].

As mentioned earlier, there was also non-participation among cases. If smoking was related to a more aggressive form of breast cancer, a greater proportion of cases who smoked may have been excluded by physicians for medical reasons, resulting in a bias toward the null. However, the overall percentage of cases diagnosed at advanced stages was small, and stage distribution was similar across participants and non-participants. Alternatively, the null associations with breast cancer risk observed for quantitative measures of active smoking and secondhand smoke exposure may be due to negative confounding by unmeasured factors, although it is difficult to speculate what these confounders could be. Non-differential misclassification of tobacco smoke exposures could have also biased the results toward the null if both the cases and the controls had equal difficulty recalling their lifetime exposures to tobacco smoke.

## Conclusion

In conclusion, exposure to tobacco smoke, either through active or secondhand smoking, was not associated with breast cancer risk in this Delaware population. Women should still be encouraged to avoid tobacco smoke exposure to prevent other adverse health effects associated with both active and secondhand smoking.

## Competing interests

The authors declare that they have no competing interests.

## Authors' contributions

DER participated in the coordination of the study, performed the statistical analyses and drafted the manuscript. RCB and HLH participated in the design of the study and helped to draft the manuscript. CJN conceived of the study, participated in its design and coordination, and helped to draft the manuscript. All authors read and approved the final manuscript.

## Pre-publication history

The pre-publication history for this paper can be accessed here:



## Supplementary Material

Additional file 1 Secondhand smoke exposure at home among non-smoking breast cancer cases and controls from Delaware. Table. Click here for file
