# Supplementary material for: Case-control study of tobacco smoke exposure and breast cancer risk in Delaware
Source: BMC Cancer. 2008 Jun 2;8:157. doi: 10.1186/1471-2407-8-157 (PMC2424067; doi:10.1186/1471-2407-8-157)
Supplement: Additional file 1 — Secondhand smoke exposure at home among non-smoking breast cancer cases and controls from Delaware. Table. [file 1471-2407-8-157-S1.doc]

Additional file 1. Secondhand smoke exposure at home among non-smoking breast cancer cases and controls from Delaware

| Residential secondhand | | Childhood exposure (<18 years of age) | | | | | | | Residential secondhand | Lifetime exposure | | | | | | | |
| --- | --- | --- | --- | --- | --- | --- | --- | --- | --- | --- | --- | --- | --- | --- | --- | --- | --- |
| smoke exposure among | | cases | |  | controls | |  |  | smoke exposure among | cases | |  | | controls | |  |  |
| never-active smokers | | n | % |  | n | % | OR^a^ | 95% CI^a^ | never-active smokers | n | % |  | | n | % | OR^a^ | 95% CI^a^ |
| No residential secondhand smoke  exposure (SSE)^b^ | | 42 | 34.1 |  | 48 | 30.0 | 1.00 | reference | No residential secondhand smoke  exposure^b^ | 23 | 18.9 |  | | 32 | 20.0 | 1.00 | reference |
| Any residential SSE | | 81 | 65.9 |  | 112 | 70.0 | 0.81 | 0.47-1.40 | Any residential SSE | 99 | 81.1 |  | | 128 | 80.0 | 1.06 | 0.56-2.02 |
|  | |  |  |  |  |  |  |  |  |  |  |  | |  |  |  |  |
| Total childhood years^c^ | |  |  |  |  |  |  |  | Total childhood years | |  |  | |  |  |  |  |
| 1-8 | | 7 | 5.7 |  | 11 | 6.9 | 0.89 | 0.30-2.67 | 1-17 | 22 | 18.0 |  | | 28 | 17.5 | 1.30 | 0.57-2.98 |
| 9-17 | | 18 | 14.6 |  | 24 | 15.4 | 0.90 | 0.41-1.97 | 18-20 | 32 | 26.2 |  | | 39 | 24.4 | 1.04 | 0.49-2.25 |
| 18 | | 56 | 45.5 |  | 77 | 48.4 | 0.78 | 0.43-1.39 | 21-35 | 17 | 13.9 |  | | 29 | 18.1 | 0.79 | 0.33-1.87 |
|  | |  |  |  |  |  |  |  | 36+ | 28 | 33.0 |  | | 32 | 20.0 | 1.11 | 0.50-2.46 |
|  | |  |  |  |  |  |  |  |  |  |  |  | |  |  |  |  |
| Total smoker-years^c^ | |  |  |  |  |  |  |  | Total smoker-years^c^ |  |  |  | |  |  |  |  |
| 1-17 | | 17 | 13.8 |  | 29 | 18.2 | 0.75 | 0.35-1.60 | 1-18 | 35 | 28.7 |  | | 39 | 24.4 | 1.24 | 0.58-2.64 |
| 18 | | 41 | 33.3 |  | 45 | 28.3 | 0.91 | 0.48-1.71 | 19-26 | 8 | 6.6 |  | | 24 | 15.0 | 0.44 | 0.16-1.27 |
| 19+ | | 23 | 18.7 |  | 38 | 23.9 | 0.73 | 0.36-1.51 | 27-41 | 32 | 26.2 |  | | 33 | 20.6 | 1.33 | 0.62-2.88 |
|  | |  |  |  |  |  |  |  | 42+ | 24 | 19.7 |  | | 32 | 20.0 | 1.01 | 0.45-2.27 |
|  | |  |  |  |  |  |  |  |  |  |  |  | |  |  |  |  |
| Pack-years (cigarettes only) | | |  |  |  |  |  |  | Pack-years (cigarettes only)^e^ | |  |  | |  |  |  |  |
| 1-14 | 9 | | 9.7 |  | 24 | 18.1 | 0.43 | 0.17-1.08 | 1-15 | 11 | 12.4 |  | | 25 | 18.7 | 0.56 | 0.22-1.45 |
| 15-18 | 12 | | 12.9 |  | 19 | 14.3 | 0.76 | 0.31-1.84 | 16-27 | 12 | 13.5 |  | | 24 | 17.9 | 0.59 | 0.23-1.53 |
| 19-35 | 11 | | 11.8 |  | 15 | 11.3 | 0.88 | 0.35-2.25 | 28-44 | 24 | 27.0 |  | | 26 | 19.4 | 1.42 | 0.61-3.30 |
| 36+ | 19 | | 20.4 |  | 27 | 20.3 | 0.76 | 0.34-1.69 | 45+ | 19 | 21.4 |  | | 27 | 20.2 | 0.93 | 0.40-2.19 |
|  |  | |  |  |  |  |  |  |  |  |  |  | |  |  |  |  |
| Weighted pack-years (cigarettes only)^d^ | | | |  |  |  |  |  | Weighted pack-years (cigarettes only)^d^ | | | |  | |  |  |  |
| 1-2 | 15 | | 16.1 |  | 23 | 17.6 | 0.76 | 0.33-1.76 | 1-2 | 14 | 15.9 |  | | 26 | 19.7 | 0.64 | 0.25-1.60 |
| 3-5 | 12 | | 12.9 |  | 23 | 17.6 | 0.58 | 0.25-1.39 | 3-8 | 22 | 14.8 |  | | 29 | 16.7 | 1.05 | 0.46-2.41 |
| 6-10 | 12 | | 12.9 |  | 18 | 13.7 | 0.80 | 0.32-1.98 | 9-15 | 16 | 25.0 |  | | 23 | 22.0 | 1.01 | 0.41-2.50 |
| 11+ | 12 | | 12.9 |  | 19 | 14.5 | 0.73 | 0.29-1.83 | 16+ | 13 | 18.2 |  | | 22 | 17.4 | 0.81 | 0.32-2.05 |

^a^ Adjusted for age, menopausal status, body mass index (<25, 25-29, 30+), age at menarche (<12 vs. 12+), age at first live birth (nulliparous, <30, 30+), oral contraceptive use (ever vs. never), other hormone use (ever vs. never), family history of breast cancer (yes vs. no), alcohol consumption (ever drank 12 drinks in lifetime vs. had 12 drinks in lifetime but never had 1+ drink per month for 6+ months vs. ever had 1+ drink per month for 6+ months); data for adjustment factors was missing for 4 cases and 3 controls

^b^ All data on residential secondhand smoke exposures were missing for childhood for 1 case and 1 control and for lifetime for 1 case and 1 control

^c^ 9 cases/17 controls and 6 cases/8 controls were exposed only to tobacco products other than cigarettes before age 18 and in their lifetime, respectively, and therefore contributed to total years, total smoker-years, but not to the cigarette-specific measures

^d^ 0 cases/2 controls and 1 case/2 controls were missing data on hours of exposure per day in childhood or lifetime, respectively, and did not contribute to weighted pack-years

^e^ 27 cases/18 controls were missing data on packs/day of exposure either in childhood and/or adulthood and contributed only to years and smoker-years
